# Psychological risk factors that predict social networking addiction in students

**DOI:** 10.1192/j.eurpsy.2024.526

**Published:** 2024-08-27

**Authors:** W. Abid, F. Guermazi, M. Mnif, D. Mnif, I. Feki, I. Baati, J. Masmoudi

**Affiliations:** ^1^psychiatry A department, Hedi Chaker university hospital, Sfax, Tunisia

## Abstract

**Introduction:**

Social networks (SN) addiction is a serious problem among young adults that requires increased attention.

**Objectives:**

The aim of the study was to assess the relationships between internet addiction and selected psychological characteristics of university students.

**Methods:**

This is a descriptive and analytical study, conducted over the period from November 2022 to January 2023, among students in various fields. SN addiction was measured using the Social Media Addiction Scale-Student Form (SMAS-SF). The Rosenberg scale was used to assess global self-esteem and the Social Self-Esteem Inventory was used to assess social self-esteem. The Big Five Personality-10 (BFI 10) scale was used to assess the 5 personality dimensions.

**Results:**

A total of 116 students, with an average age of 25.49, took part in the study. Most students (91.4%) were over 20 years old. They were female in 78.4% of cases. They enrolled in postgraduate studies in 55.2% of cases. The majority of students (59,5%) studied medicine. According to the SMAS-SF scale, the average score was 75.87. The mean score for social self-esteem score was 122.03. Sixty-four participants (55.2%) had low and very low self-esteem. The dominant personality dimensions were extraversion and neuroticism in 15.5% each. Addiction to SN was significantly associated with very low global self-esteem (p=0.028) and a lower social self-esteem score (p=0.011). Low conscientiousness and neuroticism were significantly associated with increased SN use (p=0.007, p=0.004 respectively).

**Conclusions:**

This study provides a better understanding of the phenomenon of addiction to SN, and enables us to tailor prevention and care more effectively. The psychological factors associated with this behavior need to be more explored in future research.

**Disclosure of Interest:**

None Declared

